# Monetary policy, debt maturity structure and corporate investment efficiency: Evidence from China

**DOI:** 10.1371/journal.pone.0328358

**Published:** 2025-08-01

**Authors:** Liping Zheng, Guangzhen Liu, Ziwei Liang, Chengyi Liu

**Affiliations:** Finance and Economics College, Jimei University, Xiamen, China; Yamanashi Gakuin University: Yamanashi Gakuin Daigaku, JAPAN

## Abstract

This paper examines the impact of monetary policy on corporate investment efficiency from the perspective of debt maturity structure by selecting data on Chinese non-financial listed firms and Chinese macroeconomic data from 2007–2022. The results find that loose monetary policy can have a dual effect on corporate investment efficiency by extending corporate debt maturity structure, which is manifested in alleviating corporate under-investment and promoting corporate over-investment. Heterogeneity analysis shows that in high bank competition areas, the debt maturity structure effect of monetary policy is effective in mitigating under-investment and promoting over-investment by enterprises. In contrast, in low bank competition regions, the debt maturity structure effect of monetary policy promotes corporate over-investment but has no significant effect on corporate under-investment. In addition, the debt maturity structure effect of monetary policy can effectively alleviate under-investment and promote over-investment of enterprises with low financing constraints. And it has no significant effect on the under-investment and over-investment behavior of high financing constraint enterprises. The research in this paper reveals the specific mechanism of monetary policy affecting the investment efficiency of enterprises, which has certain reference value for the objective evaluation of monetary policy effects.

## 1. Introduction

Enterprises are the cornerstone of a country’s economy, and investment is the main way to achieve their growth. Investment creates wealth for shareholders through resource allocation. Although under the perfect market hypothesis, firms’ investment behavior is not limited by financing constraints and is only related to the investment opportunities they face [1]. However, in reality, the actual investment level of enterprises often deviates from the optimal investment level, resulting in low investment efficiency of enterprises. This inefficient investment not only reduces the value of enterprises at the micro level, but also affects the resource allocation efficiency of the entire economy at the macro level, thereby inhibiting the sustained and healthy development of the macroeconomy [2].

Investment inefficiency is a widespread problem in global economies, which not only leads to a decline in total factor productivity, but even threatens a country’s long-term economic growth potential and national security. Specifically, the Japanese government has continued to provide excessive investment support to inefficient firms through distortionary economic policies, exacerbating resource mismatch and ultimately leading to a significant decline in total factor productivity [3]. Under-investment in the United Kingdom accelerated the deindustrialization process [4]. Russia’s under-investment has led to weak economic growth [5]. And the under-investment in the United States has threatened national security [6]. China, as the world’s largest emerging market country, has always had investment as one of the three main drivers of its economic growth..China’s economy is currently in a new stage of transformation and upgrading. Optimizing corporate investment structure and improving corporate investment efficiency are the micro-foundations for high-quality economic development. Therefore, we are concerned about the investment efficiency of Chinese companies.

Existing literature believes that financing constraints, agency problems caused by information asymmetry, imperfect financial systems, and imperfect management systems are the main reasons for the inefficiency of corporate investment [2,7–10]. Existing studies have examined the influencing factors of corporate investment efficiency from both internal and external dimensions. Firms’ internal factors such as financing constraints [8], managerial overconfidence [11], board tenure diversity [12], ESG performance [13], and corporate digitalization level [9]. External factors focus on the financial system [14], monetary policy [15], the degree of improvement of the social credit system [16], and the level of digital financial development [10], etc.

Monetary policy, as one of the important tools of macro-control, can have an impact on the financing constraints and agency problems of enterprises through a variety of channels such as credit channels, interest rate channels, and expectation management channels [15,17–19]. Monetary policy is an important external factor affecting the efficiency of corporate investment. Riccetti *et al.* [18] find that overfinancing increases the likelihood of over-investment when the monetary environment is looser. Li *et al.* [19] find that increased liquidity through medium-term lending facility operations improves the investment efficiency of Chinese non-SOEs by reducing their credit constraints, agency costs, and operating risk. Yang *et al.* [2] find that tightening monetary policy exacerbates financing constraints and leads to more severe under-investment behavior of firms. Wan and Lee [15] take Chinese firms as the object of their study, focusing on the effects of the credit channel and the interest rate channel on the investment efficiency of firms, in order to test the effectiveness of the transmission mechanism of monetary policy in China. They found that increasing the money supply can effectively curb corporate under-investment and the credit channel is effective. While increasing interest rates does not effectively curb over-investment to achieve the purpose of preventing economic overheating, and the interest rate channel conduction is blocked.

It can be seen that the main analytical logic of the current literature focusing on the impact of the credit channel of monetary policy on the efficiency of corporate investment is that changes in monetary policy lead to changes in the financing environment faced by enterprises, such as the availability of credit and changes in financing costs, which in turn change corporate investment decisions, ultimately affecting the efficiency of corporate investment [2,19]. Obviously, the current research on the impact of monetary policy credit channels on corporate investment efficiency mainly focuses on the impact of changes in total credit under monetary policy adjustments on corporate investment efficiency, neglecting the impact of credit structure adjustments. Zhong *et al.* [20] found that the more restrictive the monetary policy, the shorter the maturity structure of corporate credit. Cutillas Gomariz and Sanchez Ballesta [21] argue that debt short-termization can enhance the efficiency of corporate investment by strengthening supervision and reducing information asymmetry. Therefore, can monetary policy affect the efficiency of corporate investment by changing the maturity structure of corporate debt? This is the question we want to investigate.

Here, we distinguish between the liquidity effect and the debt maturity structure effect of monetary policy. The liquidity effect of monetary policy is the result of a loose monetary policy, leading to an increase in total credit and a relatively abundant market liquidity. The debt maturity structure effect of monetary policy is characterized by an increase in long-term credit, improving the term structure of corporate credit when monetary policy is loose [22]. There are differences in the mechanism of action between the debt maturity structure effect of monetary policy and the liquidity effect of monetary policy. The monetary policy liquidity effect affects corporate investment efficiency by easing corporate financing constraints in aggregate terms. Monetary policy debt maturity structure effect is from the perspective of enhancing investment and financing maturity matching to affect corporate investment efficiency. Because based on the investment and financing maturity matching theory, when the financing and investment project maturity is more matched, it can better promote investment and enhance efficiency. Therefore, this paper focuses on the effect of monetary policy debt maturity structure effect on enterprise investment efficiency from a new perspective.

This paper based on the data of China’s A-share listed companies from 2007 to 2022. Empirical results show that loose monetary policy has a dual effect on corporate investment efficiency. On the one hand, loose monetary policy suppresses corporate under-investment and improves corporate investment efficiency. On the other hand, loose monetary policy promotes corporate over-investment and reduces corporate investment efficiency. Among them, through the test of the mediating effect, it is found that loose monetary policy suppresses corporate under-investment and promotes corporate over-investment by extending the debt maturity structure of enterprises, and the monetary debt maturity structure channel is unblocked. Further, heterogeneity analysis shows that in high bank competition areas, the debt maturity structure effect of monetary policy is effective in mitigating under-investment and promoting over-investment by enterprises. In contrast, in low bank competition regions, the debt maturity structure effect of monetary policy promotes corporate over-investment but has no significant effect on corporate under-investment. In addition, the debt maturity structure effect of monetary policy can effectively alleviate under-investment and promote over-investment of enterprises with low financing constraints. And it has no significant effect on the under-investment and over-investment behavior of high financing constraint enterprises.

The possible contributions are: first, the innovation of research perspective. We study the impact of monetary policy on corporate investment efficiency from the perspective of the debt maturity structure effect of monetary policy, arguing that monetary policy affects corporate investment efficiency by changing corporate debt maturity knots. In contrast, the existing literature is mostly based on the perspective of the liquidity effect of monetary policy, which believes that monetary policy affects corporate investment efficiency by changing the volume of financing [15,19]. This paper enriches the analysis of the mechanism by which monetary policy affects corporate investment efficiency.

Second, the innovation of research findings. Existing studies have argued that debt short-termization can enhance firm efficiency by strengthening supervision and reducing information asymmetry [21]. In contrast, this paper finds that the effect of debt maturity structure on firms’ investment efficiency is two-sided. A longer debt maturity structure mitigates corporate under-investment and at the same time exacerbates over-investment.

Third, the relevant research findings provide reference for the formulation of monetary policy. The heterogeneity results indicate that the effect of aggregate monetary policy is affected by the characteristics of banks and enterprises themselves. The transmission process of monetary policy may be distorted. For example, this paper finds that loose monetary policy further exacerbates over-investment by firms with low financing constraints, while failing to alleviate under-investment by firms with high financing constraints. Therefore an important practical inspiration of this paper is to implement a targeted structural monetary policy to provide financial support in a targeted manner in order to better serve the real economy. This paper provides theoretical support for the implementation of structural monetary policy.

The rest of the paper is organized as follows: section 2 presents the Literature review and hypotheses development. Section 3 describes the model setting, variable measures and data sources. Section 4 reports the results of the empirical tests. Section 5 presents the research conclusions and policy recommendations.

## 2. Literature review and hypotheses development

### 2.1 The impact of monetary policy on the debt maturity structure of corporate

Theoretically, the enterprise debt maturity structure is jointly decided by both the capital supplier (banks and other financial institutions) and the capital demander (enterprises). Analyzing from the firm’s perspective, five existing theories explain the choice of corporate debt maturity structure: agency cost theory [7,23,24], maturity matching theory [25], tax theory, signaling theory [26], and liquidation risk theory [27]. Analyzing from the perspective of capital supply, the maturity structure of corporate debt is influenced by the willingness and ability of financial institutions to supply capital. Existing studies have focused on the impact of factors such as firm traits [28], taxation [29], corporate governance [30], and macroeconomics [31] on the maturity structure of corporate debt.

Similarly, we can comprehensively analyze the impact of monetary policy on the maturity structure of corporate debt from the perspectives of corporate capital demand and bank capital supply. From a corporate demand perspective, first of all, in a loose monetary policy environment, the cost of capital decreases, the expectation of economic growth improves, and long-term corporate investment increases. Maturity matching theory suggests that matching the maturity of debt and assets will reduce agency costs for firms and that debt maturity should remain positively correlated with asset maturity. Based on the theory of term matching, corporations increase their demand for long-term funds in order to match the terms of their assets. Secondly, signaling theory suggests that a firm’s choice of financing structure has a signaling effect. High-quality firms signal to the market that they are high-quality firms by choosing short-term debt with less pricing deviation. The theory of information asymmetry suggests that in a loose monetary policy environment, the asymmetry between lenders and borrowers decreases due to the improved stability of corporate operations. As a result, the incentive for high-quality firms to choose short-term credit for signaling purposes is weakened. Corporations weaken their preference for short-term loans, and choose the appropriate credit term based on operational needs more often. Finally, liquidation risk theory suggests that there is a risk of liquidation if a firm fails to pay its debts in a timely manner, so firms with high liquidation risk will take advantage of the favorable financing environment during the period of loose monetary policy to obtain more low-cost long-term loans.

**Fig 1:**
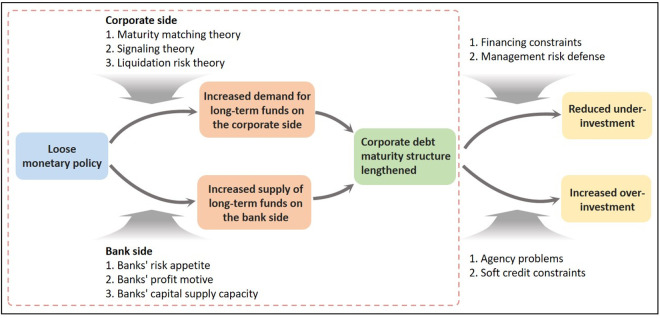
Theoretical model diagram.

From the perspective of capital supply, while a firm’s debt maturity structure is typically influenced by both the firm’s internal demand and the external capital supply, the supply of capital has a greater impact on the maturity structure of a firm’s debt under the constraint of limited loanable capital. Zhong *et al.* [20] argue that the maturity structure of enterprise credit is primarily shaped by the maturity structure of the available fund supply. In the real environment of China’s lagging bond market development, the main suppliers of corporate credit are banks. When deciding the credit term, banks must consider risk and return comprehensively and realize the balance between return and risk by adjusting the term of the loans issued. From the perspective of risk management, on the one hand, the low interest rate of loose monetary policy pushes up the value of assets and collateral from the asset price channel, and enhances the risk tolerance of banks [32]; on the other hand, it brings about the decline of corporate finance costs and the increase of profits and cash flow, which reduces the bank’s risk perception of long-term loans, and thus the loose monetary policy prompts banks to increase the proportion of long-term credit [33]. From a profit-driven perspective, based on the asset substitution effect, although the low interest rate policy has led to a decline in both long and short-term lending rates, most of the time, long-term rates are higher than short-term ones, and banks tend to replace short-term loans with long-term ones. Based on the perspective of capital supply capacity, the ability of financial institutions to provide long-term capital is affected by the economic environment, and banks have a stronger ability to supply long-term credit in an accommodative monetary policy environment. Therefore, in an accommodative monetary policy environment, banks are more able and willing to provide long-term funding, which in turn enhances the debt maturity structure of firms. A simplified theoretical analysis process can be seen in the dashed box in the left half of Fig 1. Loose monetary policy lengthens the debt maturity structure through both the supply and demand channels of funds. Thus, our first hypothesis is as follows:

Hypothesis 1: Monetary policy has a debt maturity structure effect, and loose monetary policy can effectively extend the debt maturity structure of enterprise.

### 2.2 The impact of monetary policy debt maturity structure effect on corporate investment efficiency

Enterprise investment inefficiency behavior is mainly manifested as under-investment and over-investment. We analyze the impact of the debt maturity structure effect of monetary policy on the causes of corporate under-investment and over-investment to determine the effect of monetary policy on corporate under-investment and over-investment behavior.

In terms of the causes of under-investment, first, financing constraints are an important cause of under-investment by firms [2]. Yang *et al.* [2]found that tight monetary policy exacerbates financing constraints and leads to more severe under-investment behavior. It is worth noting that the financing constraints faced by firms are not only manifested in the lack of total debt, but also in the mismatch between debt maturity and investment horizon. It is worth noting that the financing constraints faced by firms are not only manifested in a shortage of total debt. The mismatch between debt maturity and investment maturity can also impose constraints on firms’ investment. Due to the lack of long-term funding support that matches physical investment, enterprises are forced to abandon investment projects with positive net cash flows, leading to investment under-investment. Under the loose monetary policy environment, firms are more likely to obtain long-term debt support [20], and the match between the maturity structure of firms’ debt and their investment maturity rises, which can promote firms’ investment and alleviate their under-investment. Second, management risk defense is another important reason that inhibits firms’ investment behavior and contributes to under-investment.. If long-term investment is to be made, enterprises may face the risk of unstable cash flow. In order to avoid the risks associated with investment and financing maturity mismatches, risk-averse managers may forego investments with positive net present value for reputational and other reasons. Li *et al.* [34] found that managerial defense significantly diminished firms’ R&D investment.Under a loose monetary policy environment, enterprises are more likely to obtain long-term credit support, the match between the corporate debt maturity structure and the investment maturity rises. The risk of enterprises making long-term investments is reduced, which can alleviate the worries of risk-averse managers about investment, promote corporate investment, and alleviate the under-investment of enterprises. The simplified theoretical analysis process can be seen in the upper right part of Fig 1. Summarizing the above analysis, we propose Hypothesis 2:

Hypothesis 2: The debt maturity structure effect of monetary policy has a significant impact on the efficiency of corporate investment, and loose monetary policy mitigating firms’ under-investment behavior by lengthening their debt maturity structure.

From the perspective of the causes of corporate over-investment, agency problems and soft credit constraints are the main reasons for corporate over-investment [8,35]. Based on the rational person assumption, due to the inconsistency of interests between the principal and the agent and the problem of information asymmetry between the two, the management will over-invest out of the selfish motive of improving reputation or wages [36]. Further, we relax the assumption of rational people and consider the irrational situation of the management. Overconfident management with an optimistic estimation of project prospects and risk control ability will not only overestimate the future cash flow but also underestimate the risk of the project, resulting in over-investment. Ben-David *et al.* [37] found that over-confident managers tend to invest more, take on more debt, and invest in long-term rather than short-term projects. Short-term debt has a stronger monitoring role [38], and a shorter debt maturity structure reduces over-investment [21]. Long-term loans, due to their long maturity, are a weaker check on firms’ over-investment behavior. When firms obtain more long-term loans, the pressure from short-term cash flows is reduced, and overconfident managers are likely to invest in more long-term projects with negative net cash flows, exacerbating the extent of firms’ over-investment. Han and Zhao [39] found that the shortening of the debt maturity structure can alleviate the agency conflict between shareholders and management of listed companies in our country and curb over-investment behavior. A longer-term debt maturity structure may therefore further exacerbate corporate over-investment. In addition, there is a problem of soft budget constraints in the Chinese credit market. Banks provide sufficient investment funds for government-supported enterprises, like state-owned enterprises and industry policy-supported enterprises, which often results in over-investment [35]. Under the loose monetary policy environment, enterprises with the resource endowment advantages mentioned above are more likely to obtain long-term loans, and when enterprises have more long-term funds and their free cash flow is more abundant, agency conflicts will be further amplified, which may promote over-investment behavior. The simplified theoretical analysis process can be seen in the lower right part of Fig 1. Summarizing the above analysis, we propose Hypothesis 3:

Hypothesis 3: The debt maturity structure effect of monetary policy has a significant impact on the efficiency of corporate investment, and loose monetary policy can promote corporate over-investment behavior by extending the debt maturity structure of firms.

### 2.3 The heterogeneous impact of bank competition

Based on the above analysis, loose monetary policy affects the ability and willingness to supply long-term credit by enhancing banks’ risk appetite, which in turn affects the debt maturity structure of firms and ultimately acts on the efficiency of corporate investment. The degree of regional bank competition is an important factor affecting banks’ risk appetite. A higher degree of bank competition will force banks to relax their credit standards in order to increase profits and meet capital market expectations, and banks will be more likely to provide more firms with long-term loans with a higher risk of default. Bank competition can be superimposed on loose monetary policy, amplifying the incentive effect of loose monetary policy on bank risk-taking. Thus in regions where bank competition is more intense, the boost to banks’ risk appetite from loose monetary policy is greater, and new bank funding will cover both overinvested and underinvested firms. The debt maturity structure channel of monetary policy can play a full role in enhancing the debt maturity structure of underinvested and overinvested firms, reducing firms’ under-investment and reinforcing firms’ over-investment. In contrast, in regions where banks are less competitive, accommodative monetary policy has less of an effect in enhancing banks’ risk appetite. Banks will favor giving long-term funding to firms with over-investment behavior over underinvested firms. This is because overinvested firms tend to have greater financial strength and social connections, and it is less risky to extend loans to them. As a result, the debt maturity structure channel of monetary policy can play only a limited role in areas where banks are less competitive, potentially promoting over-investment but having no significant effect on under-investment. This leads to hypothesis 4:

Hypothesis 4: The debt maturity structure effect of monetary policy mitigates under-investment and promotes over-investment in areas with high bank competition, while the debt maturity structure effect of monetary policy promotes over-investment but has no significant effect on under-investment in areas with low bank competition.

### 2.4 The heterogeneous impact of financing constraints

Financing constraints are one of the important reasons affecting the investment efficiency of enterprises, financing constraints will limit the investment flexibility and scale of enterprises, resulting in lower investment efficiency. Based on the previous analysis, loose monetary policy mitigates corporate under-investment and promotes corporate over-investment by lengthening the debt maturity structure of enterprises. If the above mechanism is valid, for enterprises facing high financing constraints, the lengthening of the debt maturity structure in an easy monetary policy environment cannot solve the problem of aggregate underfunding. Therefore, for the under-investment and over-investment behavior of enterprises with high financing constraints, the debt maturity structure effect of monetary policy is relatively insignificant. For enterprises facing smaller financing constraints, further lengthening of their debt maturity structure under the condition of relatively abundant funds makes the maturity of their funds better match the maturity of their investment projects, which in turn can stimulate enterprise investment. Therefore the debt maturity structure effect of monetary policy will effectively alleviate under-investment by low financing constraint enterprises, and at the same time will stimulate further over-investment by low financing constraint enterprises. This leads to hypothesis 5:

Hypothesis 5: The debt maturity structure effect of monetary policy will effectively alleviate the under-investment of enterprises with low financing constraints and promote the over-investment of enterprises with low financing constraints, while it has no significant effect on the under-investment and over-investment behavior of enterprises with high financing constraints.

## 3. Research design

### 3.1 Data and sample selection

The study selected China’s A-share listed companies from 2007 to 2022 as the research objects. The financial data and corporate governance data come from the CSMAR database, and the monetary policy data come from the website of the People’s Bank of China. Financial listed companies, ST companies, and companies with incomplete information samples are excluded, and all continuous variables are winsorized on the 1% quantile and 99% quantile.finally we obtain 29,154 company-annual samples.

### 3.2 Model specification

The model to test the effect of monetary policy on the maturity structure of corporate debt is set up as follows:


$$Ldebt_{it}=\alpha_{0}+aMP_{t}+\varphi Controls_{it}+\gamma_{i}+\sum Industry+\epsilon_{it}$$ (1)

Further, drawing on the studies of Judd and Kenny [40], Baron and Kenny [41], and Wen *et al.* [42], a mediated effects model is used to examine the effects and mechanisms of monetary policy on the investment efficiency of firms. Judd and Kenny [40] pioneered the introduction of mediation effect analysis into social science program evaluation. Baron and Kenny [41], Wen *et al.*[42] clarified the modeling and testing process of mediation effect. The mediation effect test is completed by three steps, firstly, testing the effect of the independent variable on the dependent variable, secondly, testing the effect of the independent variable on the mediator variable, and finally, testing the effect of the independent variable and the mediator variable on the dependent variable. Zheng *et al.* [43] used the mediation effect model to find that loose monetary policy suppresses the level of firms’ “short-term debt for long-term investment” behavior by prolonging the maturity structure of corporate debt. Therefore, this paper sets the following model.

Firstly, for the sample of under-investment, the effect of monetary policy on corporate under-investment is tested through model (2). secondly, the effect of monetary policy on the debt maturity structure of underinvested firms is tested through model (3). Finally, the effect of monetary policy and debt maturity structure on corporate under-investment is considered simultaneously through model (4). The three models (2)-(4) form a complete mediation effect analysis. Similarly, for the sample of over-investment, the effect of monetary policy debt maturity structure effect on corporate over-investment is tested through models (5)-(7).


$$\;Under{INV}_{it}=\alpha_{0}+b_{1}{MP}_{t}+\varphi {Controls}_{it}+\gamma_{i}+\sum Industry+\varepsilon_{it}$$ (2)

$$\;{Ldebt}_{it}=\alpha_{0}+b_{2}{MP}_{t}+\varphi {Controls}_{it}+\gamma_{i}+\sum Industry+\varepsilon_{it}$$ (3)

$$\;Under{INV}_{it}=\alpha_{0}+b_{3}{MP}_{t}+b_{4}{Ldebt}_{it}+\varphi {Controls}_{it}+\gamma_{i}+\sum Industry+\varepsilon_{it}$$ (4)

$$Over{INV}_{it}=\alpha_{0}+b_{5}{MP}_{t}+\varphi {Controls}_{it}+\gamma_{i}+\sum Industry+\varepsilon_{it}$$ (5)

$${Ldebt}_{it}=\alpha_{0}+b_{6}{MP}_{t}+\varphi {Controls}_{it}+\gamma_{i}+\sum Industry+\varepsilon_{it}$$ (6)

$$\;Over{INV}_{it}=\alpha_{0}+b_{7}{MP}_{t}+b_{8}{Ldebt}_{it}+\varphi {Controls}_{it}+\gamma_{i}+\sum Industry+\varepsilon_{it}\;$$ (7)

Where i denotes the firm, t denotes time, ${Ldebt}_{it}$ measures the debt maturity structure of firm i in year t, $Under{INV}_{it}$ denotes firm i’s under-investment in year t, ${\text{OverINV}}_{\text{it}}$ denotes firm i’s over-investment in year t, $\;{MP}_{t}$ represents the level of monetary policy in year t, ${Controls}_{it}$ is the set of control variables, $\gamma_{i}$ represents controlling for individual firm fixed constant vectors, ∑Industryrepresents the effect of controlling the industry, $\alpha_{0}$ is the intercept, $a,b_{1},b_{2},b_{4},b_{5},b_{6},b_{8}$ represent the regression coefficients of the key variable, φ is a vector of constants, and $\varepsilon_{it}$ is the random error term. Since monetary policy is a time series variable, introducing time-fixed effects directly into the model will cause covariance problems and lead to invalid estimation results, so this paper draws on the treatment of Sun *et al* [22].and does not control for time effects in the model.

### 3.3 Definition of variables

The investment inefficiency is measured with reference to the classical model of Richardson [44] as follows:


$$INV_{i,t}=\alpha_{0}+\alpha_{1}Size_{i,t-1}+\alpha_{2}Lev_{i,t-1}+\alpha_{3}Q_{i,t-1}+\alpha_{4}Cash_{i,t-1}+\alpha_{5}{Roa}_{i,t-1}\;+\;\alpha_{6}ListY_{i,t-1}+\alpha_{7}RET_{i,t-1}+\alpha_{7}INV_{i,t-1}+\varepsilon_{i,t}$$ (8)

Where $\text{IN}{\text{V}}_{\text{i,t}}$ denotes the amount of capital invested by firm i in year t, measured by the change in net value of (fixed assets + construction in progress + intangible assets + long-term investments) divided by average total assets; $Size_{i,t-1}$, $Lev_{i,t-1}\;and\;ListY_{i,t-1}$ are the logarithm of total assets, gearing ratio and length of listing of listed companies, respectively; $Q_{i,t-1}$ is the growth opportunity of firm i at the end of year t-1, measured as the sum of the total market value of equity and the book value of liabilities divided by total assets; $Cash_{i,t-1}$ is the cash holdings of firm i at the end of year t-1, measured by ending money funds divided by ending total assets; $RET_{i,t-1}$ represents the annual return rate calculated based on the monthly stock return rates from May of the year (t-1) to April of the year t, adjusted for market conditions. The residual in equation (8) reflects the deviation of actual investment from the optimal level of investment, taking its positive value to measure over-investment (OverINV) and its negative opposite to measure under-investment (UnderINV).

Monetary policy (MP) is measured using the quantitative monetary policy instrument M2 growth rate.. The debt maturity structure (Ldebt) expressed as long-term loan divided by total liabilities.

Based on Huang *et al.* [45], Hu *et al.* [46], and Wu *et al.* [47], firms’ investment efficiency and debt maturity structure are influenced by firm characteristics, corporate governance level, and macroeconomic environment. The control variables include firm size (Size), change in total bank credit (Debt_gap), leverage (Lev), profitability (Roa), net operating cash flow (CFO), CEO-duality (Dual), and the percentage of independent directors (ID), GDP growth rate (GDP). In addition, on the one hand, considering that the change of total bank credit also affects the investment efficiency and debt maturity structure of enterprises. On the other hand, the monetary policy liquidity effect affects the total bank credit, and in order to test the effect of the monetary policy term structure effect more intuitively, the change in the total bank credit (Debt_gap) is added to the control variables. Table 1 illustrates the definition of variables.

**Table 1:** Variable definitions.

Variable name	Variables symbol	Description of the variables
under-investment	UnderINV	Refer to Richardson [44]
over-investment	OverINV	Refer to Richardson [44]
Monetary policy	MP	M2 growth rate
Debt maturity structure	Ldebt	The ratio of Long-term borrowings to total liabilities
Firm size	Size	The natural logarithm of total assets
Change in total bank credit received by firms	Debt_gap	The ratio of the change in total bank credit to total assets
Leverage	Lev	The ratio of the total liabilities to total assets
Profitability	Roa	The ratio of the net profit to total assets
Net operating cash flow	CFO	The ratio of net cash flow from operating activities to total assets
CEO-duality	Dual	A dummy variable that equals one if the CEO and board Chairman are the same person, otherwise take 0.
The percentage of independent directors	ID	Number of independent directors divided by the number of all directors
GDP growth rate	GDP	National GDP growth rate

## 4. Empirical analysis

### 4.1 Descriptive statistics

The descriptive statistics of the variables are shown in Tables 2,3,4. The data show that under both measures, there are more under-invested enterprises than over-invested enterprises in the sample data, and the problem of under-investment by enterprises is more prominent. The results of descriptive statistics for the sub-sample show that the average values of debt maturity structure, credit increment, and profitability of total assets are significantly higher for overinvested firms than for underinvested firms. The longer debt maturity structure, the more credit increment, and the higher profitability of total assets provide financial support for over-investment. The results of descriptive statistics are consistent with economic intuition.

**Table 2:** Descriptive statistics.

VarName	Obs	Mean	SD	Min	Median	Max
UnderINV	17183	0.0114	0.0100	0.0002	0.0089	0.0509
OverINV	11971	0.0179	0.0196	0.0001	0.0111	0.0882
MP	29154	0.1354	0.0447	0.0817	0.1334	0.2758
Ldebt	29154	0.1159	0.1446	0.0000	0.0619	0.6534
Size	29154	22.3204	1.3391	19.2610	22.1396	25.9501
Debt_total_gap	29154	0.0199	0.1166	−0.3637	0.0054	0.4047
Lev	29154	0.0049	0.0019	0.0005	0.0050	0.0090
Roa	29154	0.0327	0.0621	−0.2424	0.0316	0.2381
CFO	29154	0.0454	0.0758	−0.8877	0.0448	0.8759
Dual	29154	0.2251	0.4176	0.0000	0.0000	1.0000
ID	29154	0.3705	0.0559	0.0000	0.3333	0.8000
GDP	29154	0.0776	0.0269	0.0219	0.0777	0.1423

**Table 3:** Descriptive statistics:under-investment sub-sample.

VarName	Obs	Mean	SD	Min	Median	Max
UnderINV	17183	0.0114	0.0100	0.0002	0.0089	0.0509
Ldebt	17183	0.1027	0.1385	0.0000	0.0464	0.6534
Size	17183	0.5048	0.5000	0.0000	1.0000	1.0000
Debt_total_gap	17183	22.2518	1.3438	19.2610	22.0704	25.9501
Lev	17183	0.0021	0.1146	−0.3637	0.0000	0.4047
Roa	17183	0.4937	0.1979	0.0533	0.4991	0.9020
CFO	17183	0.0245	0.0663	−0.2424	0.0266	0.2381
Dual	17183	0.0436	0.0793	−0.8877	0.0432	0.8759
ID	17183	0.2094	0.4069	0.0000	0.0000	1.0000

**Table 4:** Descriptive statistics: over-investment sub-sample.

VarName	Obs	Mean	SD	Min	Median	Max
OverINV	11971	0.0179	0.0196	0.0001	0.0111	0.0882
Ldebt	11971	0.1349	0.1510	0.0000	0.0860	0.6534
Size	11971	22.4189	1.3262	19.2610	22.2458	25.9501
Debt_total_gap	11971	0.0454	0.1147	−0.3637	0.0268	0.4047
Lev	11971	0.4836	0.1827	0.0533	0.4939	0.9020
Roa	11971	0.0446	0.0534	−0.2424	0.0396	0.2381
CFO	11971	0.0480	0.0704	−0.4700	0.0465	0.6641
Dual	11971	0.2476	0.4316	0.0000	0.0000	1.0000
ID	11971	0.3710	0.0552	0.0000	0.3333	0.8000

### 4.2 Empirical results

#### 4.2.1 The effect of monetary policy on corporate debt maturity structure.

This paper first verifies the effect of monetary policy on the debt maturity structure of firms, and the results in column (1) of Table 5 show that the regression coefficient of monetary policy on the maturity structure of firms’ debt is 0.2406, indicating that for every 1% increase in the growth rate of monetary policy M2, the share of firms’ long-term borrowing in total liabilities will increase by 0.2406% on average. Loose monetary policy effectively extends the debt maturity structure of firms,. In an accommodative monetary policy environment, firms’ access to long-term loans has increased and their debt maturity structure has lengthened. Hypothesis 1 holds.

**Table 5:** The effect of monetary policy on corporate investment efficiency.

	full sample	Under-investment			Over-investment		
	(1)	(2)	(3)	(4)	(5)	(6)	(7)
	Ldebt	UnderINV	Ldebt	UnderINV	OverINV	Ldebt	OverINV
MP	0.2406***	−0.0077***	0.1892***	−0.0073***	0.0213***	0.3009***	0.0114*
	(0.0249)	(0.0024)	(0.0279)	(0.0024)	(0.0063)	(0.0402)	(0.0062)
Ldebt				−0.0021**			0.0329***
				(0.0011)			(0.0025)
Size	0.0263***	−0.0023***	0.0284***	−0.0023***	0.0011**	0.0202***	0.0004
	(0.0022)	(0.0002)	(0.0025)	(0.0002)	(0.0005)	(0.0031)	(0.0005)
Debt_gap	−0.1288***	−0.0103***	−0.1290***	−0.0106***	0.0303***	−0.1702***	0.0359***
	(0.0061)	(0.0008)	(0.0081)	(0.0008)	(0.0021)	(0.0099)	(0.0021)
Lev	31.4599	0.5706	24.4605	0.6226	1.4780	46.7346	−0.0588
	(2.3852)	(0.1952)	(2.6357)	(0.1969)	(0.5168)	(3.7074)	(0.5056)
Roa	0.1190***	−0.0201***	0.0608***	−0.0200***	0.0315***	0.1074***	0.0280***
	(0.0177)	(0.0023)	(0.0194)	(0.0023)	(0.0056)	(0.0324)	(0.0055)
CFO	−0.1540***	−0.0034**	−0.1329***	−0.0036***	0.0163***	−0.1906***	0.0226***
	(0.0126)	(0.0014)	(0.0146)	(0.0014)	(0.0037)	(0.0220)	(0.0036)
Dual	−0.0056**	−0.0003	−0.0069**	−0.0003	−0.0007	−0.0068	−0.0004
	(0.0028)	(0.0003)	(0.0033)	(0.0003)	(0.0007)	(0.0042)	(0.0007)
ID	−0.0027	0.0024	0.0063	0.0024	−0.0063	−0.0485	−0.0047
	(0.0231)	(0.0021)	(0.0249)	(0.0021)	(0.0055)	(0.0384)	(0.0053)
GDP	0.1484***	0.0190***	0.1045**	0.0193***	0.0706***	0.0936	0.0675***
	(0.0396)	(0.0045)	(0.0472)	(0.0045)	(0.0105)	(0.0612)	(0.0103)
_cons	−0.5984***	0.0586***	−0.6133***	0.0573***	−0.0240*	−0.4865***	−0.0080
	(0.0562)	(0.0048)	(0.0645)	(0.0049)	(0.0123)	(0.0756)	(0.0121)
Firm	Yes	Yes	Yes	Yes	Yes	Yes	Yes
Industry	Yes	Yes	Yes	Yes	Yes	Yes	Yes
Sobel test Z		2.04			8.74		
Bootstrap test 95% CI		[-0.0008, -0.0000]^a^			[0.0075, 0.0133]		
N	29154	17183	17183	17183	11971	11971	11971
Adj_R^2^	0.0975	0.0892	0.0986	0.0896	0.0580	0.1234	0.0861

Note(s): *p < 0.1; **p < 0.05; ***p < 0.01.Robust standard errors in parentheses. Robust standard errors in parentheses. ^a^All coefficients and confidence intervals are reported to four decimal places. The 95% confidence interval for the underinvestment indirect effect appears to include zero due to rounding, but the actual interval [−0.0007954, −0.0000156] excludes zero, indicating statistical significance at the 5% level.

#### 4.2.2 The impact of monetary policy debt maturity structure effect on corporate investment efficiency.

Further, we focuse on the effect of monetary policy on corporate investment efficiency. The corresponding empirical results are presented in columns (2)-(7) of Table 5. For under-investment, the results in column (2) show that the regression coefficient of the monetary policy variable on corporate under-investment is −0.0077, which is significant at the 1% level, indicating that loose monetary policy inhibits corporate under-investment. The mediation effect model is used to identify the mechanism of the maturity structure effect of monetary policy. Column (3) shows that accommodative monetary policy lengthens the debt maturity structure of under-invested firms, and that for every 1% increase in the growth rate of monetary policy M2, the share of long-term debt of underinvested firms will increase by 0.1892% on average. The results in column (4) show that the absolute value of the monetary policy coefficient becomes smaller after considering the effect of debt maturity structure on firms’ under-investment, and the extension of debt maturity structure alleviates firms’ under-investment. Partial mediation effects hold. To more rigorously verify the robustness of the mediating effect, we employ both the Sobel test and Bootstrap method for dual validation. The Sobel test statistic is $Z=\frac{\hat{a}\hat{b}}{\sqrt{{\hat{a}}^{2}s_{b}^{2}+{{\hat{b}}^{2}s}_{a}^{2}}}$, where $\hat{a}$ and $\hat{b}$ arethe estimates of the MP coefficient in model (3) and the Ldebt coefficient in model (4), respectively, and $s_{a}$ and $s_{b}$ are the corresponding standard errors. The absolute value of the calculated Sobel test Z statistic is 2.04, which significantly exceeds the critical value of 1.96. Additionally, we conduct Bootstrap resampling with 5,000 iterations, yielding a 95% confidence interval for the indirect effects of [−0.0007954, −0.0000156]. This interval lies entirely within the negative range and excludes zero. Both statistical tests confirm that the mediating effect is statistically significant, thereby validating Hypothesis 2.

For over-invested companies, the results in column (5) show that the regression coefficient of the monetary policy variable on corporate over-investment is 0.0213, which is significant at the 1% level, indicating that loose monetary policy stimulates corporate over-investment. Using the mediation effects model to identify the mechanism of the term structure effect of monetary policy, column (6) shows that accommodative monetary policy lengthens the term structure of debt for over-investing firms. Compared to the results in column (6) and (7), the absolute value of the monetary policy coefficient becomes smaller after controlling for the effect of debt maturity structure on corporate over-investment, which suggests that some of the mediation effects are effective. This shows that a certain proportion of the promoting effect of loose monetary policy on corporate over-investment is realized through the debt maturity structure channel. The absolute value of the calculated Sobel test yields a Z statistic of 8.74, which significantly exceeds the critical value of 1.96. Furthermore, Bootstrap resampling with 5,000 iterations produces a 95% confidence interval for the mediating effect of [0.0074893, 0.0133441], which lies entirely within the positive range and excludes zero. Both statistical tests consistently confirm that the mediating effect is statistically significant, thereby validating Hypothesis 2.

### 4.3 Robustness test

#### 4.3.1 Reverse causality.

Although monetary policy is a macroeconomic variable, it can theoretically be considered exogenous. However, macroeconomic policy formulation is often based on the state of the real economy and there may be endogeneity problems of reverse causality. Therefore, we mitigate the endogeneity problem of mutual causality by lagging monetary policy by one period (MP_1). The results in Table 6 show that the regression results of lagging monetary policy by one period are consistent with the benchmark regression.

**Table 6:** Robustness test: monetary policy lagged one period.

	Under-investment			Over-investment		
	(1)	(2)	(3)	(4)	(5)	(6)
	UnderINV	Ldebt	UnderINV	OverINV	Ldebt	OverINV
MP_1	−0.0062**	0.1243***	−0.0059**	0.0149**	0.2165***	0.0080
	(0.0025)	(0.0295)	(0.0025)	(0.0074)	(0.0424)	(0.0072)
Ldebt			−0.0021*			0.0318***
			(0.0011)			(0.0028)
Size	−0.0024***	0.0256***	−0.0023***	0.0011**	0.0175***	0.0005
	(0.0002)	(0.0027)	(0.0002)	(0.0005)	(0.0035)	(0.0005)
Debt_gap	−0.0098***	−0.1289***	−0.0101***	0.0288***	−0.1733***	0.0343***
	(0.0009)	(0.0083)	(0.0009)	(0.0023)	(0.0109)	(0.0024)
Lev	0.0023**	0.1315***	0.0026**	0.0072**	0.2424***	−0.0005
	(0.0011)	(0.0149)	(0.0011)	(0.0030)	(0.0217)	(0.0029)
Roa	−0.0189***	0.0503**	−0.0188***	0.0323***	0.1244***	0.0283***
	(0.0025)	(0.0221)	(0.0025)	(0.0066)	(0.0371)	(0.0065)
CFO	−0.0031**	−0.1286***	−0.0034**	0.0143***	−0.2043***	0.0208***
	(0.0015)	(0.0154)	(0.0015)	(0.0040)	(0.0257)	(0.0040)
Dual	−0.0001	−0.0056	−0.0001	−0.0006	−0.0026	−0.0005
	(0.0003)	(0.0036)	(0.0003)	(0.0008)	(0.0049)	(0.0008)
ID	0.0015	0.0042	0.0015	−0.0094	−0.0404	−0.0081
	(0.0023)	(0.0274)	(0.0023)	(0.0064)	(0.0419)	(0.0062)
GDP	0.0206***	0.0989*	0.0208***	0.0693***	0.0299	0.0684***
	(0.0053)	(0.0531)	(0.0053)	(0.0128)	(0.0704)	(0.0125)
_cons	0.0605***	−0.5481***	0.0594***	−0.0242*	−0.4244***	−0.0107
	(0.0053)	(0.0702)	(0.0053)	(0.0137)	(0.0862)	(0.0136)
Firm	Yes	Yes	Yes	Yes	Yes	Yes
Industry	Yes	Yes	Yes	Yes	Yes	Yes
N	14709	14709	14709	9442	9442	9442
Adj_R2	0.0836	0.0934	0.0840	0.0580	0.1194	0.0846

Note(s): *p < 0.1; **p < 0.05; ***p < 0.01;Robust standard errors in parentheses.

#### 4.3.2 The problem of omitted variables.

Due to the time series nature of monetary policy, this paper did not consider time-fixed effects in the baseline regression to avoid multicollinearity. The aforementioned research in this paper has referenced numerous classic literatures and has controlled as much as possible the factors affecting the debt maturity structure of enterprises. However, due to the lack of control for time-fixed effects, some macro factors may still be omitted, which may cause estimation bias. This paper further introduces two variables, the level of financial development (Financial, Sum of market capitalization of A-shares outstanding and loan balances of financial institutions divided by GDP) and the level of fiscal expenditure (Fiscal, Fiscal expenditure divided by GDP) in each province, to control for the impact of regional financial endowment conditions and local government intervention. The results in Table 7 are consistent with the baseline regression and are significant at the 1% level.

**Table 7:** Robustness test: omitted variables.

	Under-investment			Over-investment		
	(1)	(2)	(3)	(4)	(5)	(6)
	UnderINV	Ldebt	UnderINV	OverINV	Ldebt	OverINV
MP	−0.0089***	0.1536***	−0.0084***	0.0177**	0.2206***	0.0107
	(0.0027)	(0.0290)	(0.0027)	(0.0074)	(0.0446)	(0.0072)
Ldebt			−0.0033**			0.0319***
			(0.0013)			(0.0030)
Size	−0.0024***	0.0300***	−0.0023***	0.0029***	0.0212***	0.0022***
	(0.0002)	(0.0031)	(0.0002)	(0.0007)	(0.0043)	(0.0007)
Debt_gap	−0.0105***	−0.1248***	−0.0109***	0.0311***	−0.1654***	0.0364***
	(0.0010)	(0.0092)	(0.0010)	(0.0025)	(0.0119)	(0.0025)
Lev	0.4282	29.1935	0.5259	0.6682	47.4311	−0.8439
	(0.2420)	(3.1262)	(0.2445)	(0.6762)	(4.3221)	(0.6616)
Roa	−0.0238***	0.0691***	−0.0235***	0.0280***	0.0982**	0.0248***
	(0.0029)	(0.0244)	(0.0029)	(0.0075)	(0.0406)	(0.0074)
CFO	−0.0036**	−0.1428***	−0.0041***	0.0215***	−0.1999***	0.0279***
	(0.0015)	(0.0162)	(0.0016)	(0.0043)	(0.0262)	(0.0042)
Dual	−0.0000	−0.0013	−0.0000	0.0008	−0.0047	0.0009
	(0.0004)	(0.0040)	(0.0004)	(0.0009)	(0.0055)	(0.0009)
ID	0.0034	0.0144	0.0035	−0.0037	−0.0501	−0.0021
	(0.0026)	(0.0302)	(0.0026)	(0.0066)	(0.0444)	(0.0064)
GDP	0.0475***	0.1980**	0.0481***	0.0927***	0.2131*	0.0859***
	(0.0090)	(0.0834)	(0.0090)	(0.0217)	(0.1200)	(0.0212)
Fin_p	−0.0002	−0.0012	−0.0002	−0.0001	−0.0013	−0.0001
	(0.0004)	(0.0036)	(0.0003)	(0.0009)	(0.0052)	(0.0009)
Fiscal_p	0.0160**	−0.1540**	0.0154**	−0.0407**	−0.2007*	−0.0343**
	(0.0067)	(0.0689)	(0.0066)	(0.0167)	(0.1059)	(0.0164)
_cons	0.0537***	−0.6885***	0.0514***	−0.0595***	−0.4973***	−0.0436***
	(0.0060)	(0.0746)	(0.0060)	(0.0160)	(0.1021)	(0.0157)
Firm	Yes	Yes	Yes	Yes	Yes	Yes
Industry	Yes	Yes	Yes	Yes	Yes	Yes
N	12553	12553	12553	8474	8474	8474
Adj_R^2^	0.0967	0.1071	0.0975	0.0619	0.1126	0.0878

Note(s): *p < 0.1; **p < 0.05; ***p < 0.01;Robust standard errors in parentheses.

#### 4.3.3 Placebo test.

**Fig 2:**
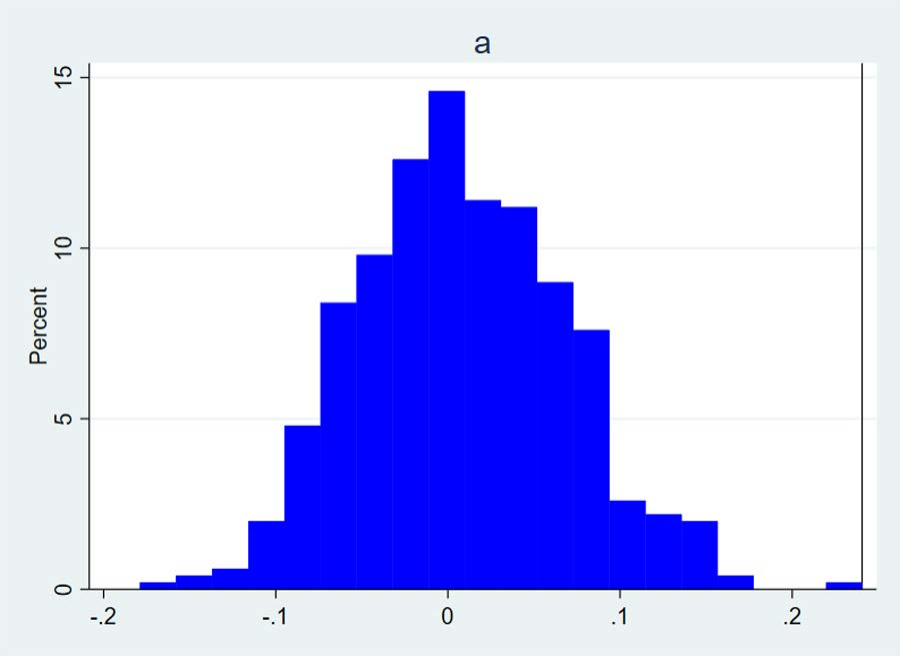
Distribution of placebo test regression coefficients a. The X-axis represents the distribution range of the placebo test coefficients for 500 trials, and the Y-axis represents the proportion of each coefficient distribution interval. The vertical line marks the position of the benchmark regression coefficient.

**Fig 3:**
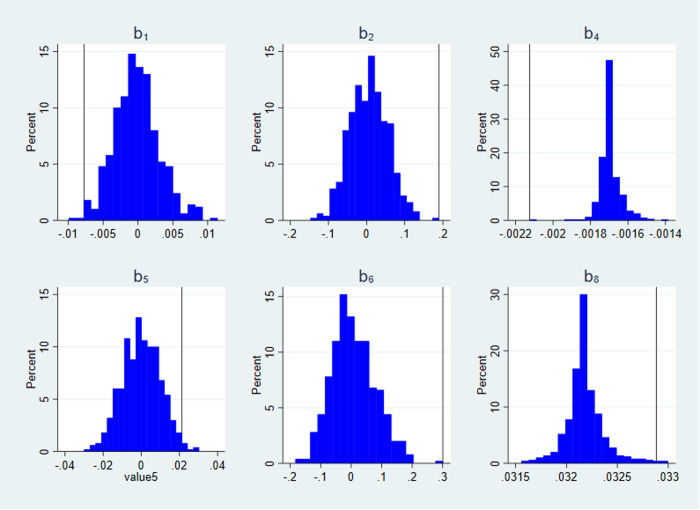
Distribution of placebo test regression coefficients ${\;b}_{1},b_{2},b_{4}{,b}_{5},b_{6},b_{8}$. The X-axis represents the distribution range of the placebo test coefficients for 500 trials, and the Y-axis represents the proportion of each coefficient distribution interval. The vertical line marks the position of the benchmark regression coefficient.

In order to test the effectiveness of monetary policy, this paper conducts a placebo test. we conduct a placebo test by forming 500 simulated sets of monetary policy data after 500 random disruptions of monetary policy. The effectiveness of monetary policy is verified by regressing the model (2)-(7) on the 500 sets of simulated monetary policy data to see whether the key regression coefficients ${\;a,b}_{1},b_{2},b_{4}{,b}_{5},b_{6},b_{8}$ in the benchmark regression fall within the range of small probability events. The results in Figs 2 and 3 show that the coefficients from the benchmark regressions fall within the extremes of the 1% range of the distribution of placebo-test coefficients. The occurrence of small probability events suggests that the impact of monetary policy on the efficiency of business investment and the monetary policy debt maturity structure channel exists.

#### 4.3.4 Alternative measure of variables.

To verify the robustness of our research findings, we conduct tests using multiple alternative measurement methods. First, we employ alternative measures for the dependent variables UnderINV and OverINV, namely UnderINV2 and OverINV2. Following Mao and Guan [48] and Biddle et al. [49], the residual in equation (9) reflects the deviation of actual investment from the optimal level of investment, taking its positive value to measure over-investment (OverINV2) and its negative opposite to measure under-investment (UnderINV2). $\text{IN}{\text{V}}_{\text{i,t}}$ and $Q_{i,t-1}$ are defined consistently with their previous definitions. Second, we also employ alternative measures for the core explanatory variable and mediating variable: the alternative measure of monetary policy MP2 is calculated as the difference between M2 growth rate and real GDP growth rate; the alternative measure of debt maturity structure Ldebt2 is measured by the proportion of long-term loans to the sum of long-term and short-term loans. The regression results in Tables 8–10 demonstrate that after replacing the main variable measurement methods, all results remain highly consistent with the baseline regressions, fully confirming the robustness of the impact of monetary policy on corporate investment efficiency.

**Table 10:** Robustness test: alternative measure of debt maturity structure.

	Under-investment			Over-investment		
	(1)	(2)	(3)	(4)	(5)	(6)
	UnderINV	Ldebt2	UnderINV	OverINV	Ldebt2	OverINV
MP	−0.0077***	0.2453***	−0.0067***	0.0213***	0.4160***	0.0162**
	(0.0024)	(0.0626)	(0.0025)	(0.0063)	(0.0776)	(0.0064)
Ldebt2			−0.0013***			0.0140***
			(0.0004)			(0.0011)
Size	−0.0023***	0.0808***	−0.0023***	0.0011**	0.0726***	0.0002
	(0.0002)	(0.0059)	(0.0002)	(0.0005)	(0.0065)	(0.0005)
Debt_gap	−0.0103***	−0.4043***	−0.0109***	0.0303***	−0.5766***	0.0391***
	(0.0008)	(0.0171)	(0.0009)	(0.0021)	(0.0211)	(0.0022)
Lev	0.5706	26.1734	0.6008	1.4780	63.0117	0.6836
	(0.1952)	(6.2640)	(0.2076)	(0.5168)	(7.1409)	(0.5266)
Roa	−0.0201***	0.2042***	−0.0203***	0.0315***	0.4378***	0.0266***
	(0.0023)	(0.0467)	(0.0023)	(0.0056)	(0.0695)	(0.0058)
CFO	−0.0034**	−0.1337***	−0.0042***	0.0163***	−0.2058***	0.0185***
	(0.0014)	(0.0340)	(0.0014)	(0.0037)	(0.0480)	(0.0037)
Dual	−0.0003	−0.0114	−0.0003	−0.0007	−0.0036	−0.0004
	(0.0003)	(0.0077)	(0.0003)	(0.0007)	(0.0097)	(0.0007)
ID	0.0024	0.0638	0.0025	−0.0063	−0.0541	−0.0053
	(0.0021)	(0.0611)	(0.0021)	(0.0055)	(0.0749)	(0.0057)
GDP	0.0190***	−0.3248***	0.0166***	0.0706***	−0.1043	0.0751***
	(0.0045)	(0.1023)	(0.0046)	(0.0105)	(0.1351)	(0.0105)
_cons	0.0586***	−1.6053***	0.0593***	−0.0240*	−1.5106***	−0.0078
	(0.0048)	(0.1448)	(0.0052)	(0.0123)	(0.1582)	(0.0125)
Firm	Yes	Yes	Yes	Yes	Yes	Yes
Industry	Yes	Yes	Yes	Yes	Yes	Yes
N	17183	15743	15743	11971	11284	11284
Adj_R^2^	0.0892	0.1427	0.0938	0.0580	0.1877	0.0841

Note(s): *p < 0.1; **p < 0.05; ***p < 0.01; Robust standard errors in parentheses.

**Table 8:** Robustness test: alternative measure of investment efficiency.

	Under-investment			Over-investment		
	(1)	(2)	(3)	(4)	(5)	(6)
	UnderINV2	Ldebt	UnderINV2	OverINV2	Ldebt	OverINV2
MP	−0.0200**	0.2024***	−0.0173**	0.1414***	0.3045***	0.0938***
	(0.0086)	(0.0269)	(0.0086)	(0.0292)	(0.0418)	(0.0283)
Ldebt			−0.0133***			0.1565***
			(0.0045)			(0.0129)
Size	−0.0051***	0.0267***	−0.0048***	0.0065***	0.0204***	0.0033
	(0.0008)	(0.0024)	(0.0008)	(0.0024)	(0.0033)	(0.0023)
Debt_gap	−0.0284***	−0.1381***	−0.0302***	0.1185***	−0.1724***	0.1454***
	(0.0031)	(0.0081)	(0.0032)	(0.0094)	(0.0104)	(0.0095)
Lev	0.0076*	0.1222***	0.0092**	0.0304**	0.2475***	−0.0083
	(0.0040)	(0.0127)	(0.0040)	(0.0132)	(0.0183)	(0.0130)
Roa	0.0428***	0.0558***	0.0435***	0.2679***	0.1437***	0.2454***
	(0.0083)	(0.0190)	(0.0083)	(0.0280)	(0.0336)	(0.0276)
CFO	−0.0289***	−0.1458***	−0.0308***	0.0154	−0.2014***	0.0469**
	(0.0060)	(0.0146)	(0.0061)	(0.0189)	(0.0231)	(0.0184)
Dual	0.0012	−0.0084***	0.0011	−0.0052	−0.0062	−0.0042
	(0.0011)	(0.0032)	(0.0011)	(0.0036)	(0.0045)	(0.0036)
ID	0.0070	0.0139	0.0071	0.0006	−0.0309	0.0054
	(0.0075)	(0.0247)	(0.0075)	(0.0253)	(0.0384)	(0.0244)
GDP	−0.0620***	0.0889*	−0.0608***	0.2711***	0.1576***	0.2464***
	(0.0155)	(0.0476)	(0.0156)	(0.0461)	(0.0593)	(0.0454)
_cons	0.1528***	−0.5998***	0.1448***	−0.1301**	−0.4372***	−0.0617
	(0.0186)	(0.0621)	(0.0187)	(0.0597)	(0.0828)	(0.0586)
Firm	Yes	Yes	Yes	Yes	Yes	Yes
Industry	Yes	Yes	Yes	Yes	Yes	Yes
N	17278	17278	17278	11034	11034	11034
Adj_R^2^	0.0301	0.1052	0.0313	0.0690	0.1324	0.0992

Note(s): *p < 0.1; **p < 0.05; ***p < 0.01;Robust standard errors in parentheses.

**Table 9:** Robustness test: alternative measure of monetary policy.

	Under-investment			Over-investment		
	(1)	(2)	(3)	(4)	(5)	(6)
	UnderINV	Ldebt	UnderINV	OverINV	Ldebt	OverINV
MP2	−0.0082***	0.1442***	−0.0079***	0.0149***	0.2315***	0.0072
	(0.0020)	(0.0212)	(0.0020)	(0.0053)	(0.0318)	(0.0052)
Ldebt			−0.0021**			0.0330***
			(0.0011)			(0.0024)
Size	−0.0023***	0.0270***	−0.0023***	0.0009*	0.0177***	0.0003
	(0.0002)	(0.0024)	(0.0002)	(0.0005)	(0.0030)	(0.0004)
Debt_gap	−0.0104***	−0.1279***	−0.0107***	0.0304***	−0.1694***	0.0360***
	(0.0008)	(0.0081)	(0.0008)	(0.0021)	(0.0099)	(0.0021)
Lev	0.5680	24.9025	0.6203	1.5242	47.2475	−0.0362
	(0.1944)	(2.6383)	(0.1962)	(0.5166)	(3.7136)	(0.5057)
Roa	−0.0202***	0.0654***	−0.0201***	0.0322***	0.1166***	0.0284***
	(0.0023)	(0.0194)	(0.0023)	(0.0056)	(0.0324)	(0.0055)
CFO	−0.0033**	−0.1337***	−0.0035**	0.0161***	−0.1934***	0.0225***
	(0.0014)	(0.0146)	(0.0014)	(0.0037)	(0.0221)	(0.0036)
Dual	−0.0003	−0.0068**	−0.0003	−0.0007	−0.0070*	−0.0005
	(0.0003)	(0.0033)	(0.0003)	(0.0007)	(0.0042)	(0.0007)
ID	0.0023	0.0059	0.0023	−0.0062	−0.0480	−0.0046
	(0.0021)	(0.0249)	(0.0021)	(0.0055)	(0.0385)	(0.0053)
GDP	0.0103**	0.2770***	0.0109**	0.0874***	0.3488***	0.0758***
	(0.0047)	(0.0575)	(0.0047)	(0.0115)	(0.0758)	(0.0113)
_cons	0.0584***	−0.5742***	0.0572***	−0.0180	−0.4160***	−0.0043
	(0.0047)	(0.0616)	(0.0047)	(0.0118)	(0.0710)	(0.0115)
Firm	Yes	Yes	Yes	Yes	Yes	Yes
Industry	Yes	Yes	Yes	Yes	Yes	Yes
N	17183	17183	17183	11971	11971	11971
Adj_R^2^	0.0897	0.0975	0.0900	0.0575	0.1215	0.0859

Note(s): *p < 0.1; **p < 0.05; ***p < 0.01;Robust standard errors in parentheses.


$${Inv}_{i,t}=\alpha_{0}+\alpha_{1}Q_{i,t-1}+\varepsilon_{i,t}$$ (9)

#### 4.3.5 Excluding the influence of the city where the enterprise is located.

Due to the prominent regional development imbalance in China, the business environment and investment opportunities in the four first-tier cities Beijing, Shanghai, Shenzhen and Guangzhou are significantly better than those in other regions, which may affect the investment efficiency of firms. Therefore, we exclude firms registered in Beijing, Shanghai, Shenzhen, and Guangzhou for robustness testing. The results in Table 11 are consistent with the benchmark regression.

**Table 11:** Robustness test: exclude firms registered in Beijing, Shanghai, Shenzhen, and Guangzhou.

	Under-investment			Over-investment		
	(1)	(2)	(3)	(4)	(5)	(6)
	UnderINV	Ldebt	UnderINV	OverINV	Ldebt	OverINV
MP	−0.0061**	0.1912***	−0.0056**	0.0222***	0.2911***	0.0118*
	(0.0027)	(0.0307)	(0.0027)	(0.0072)	(0.0434)	(0.0070)
Ldebt2			−0.0027**			0.0356***
			(0.0012)			(0.0028)
Size	−0.0024***	0.0279***	−0.0023***	0.0014***	0.0185***	0.0007
	(0.0002)	(0.0028)	(0.0002)	(0.0005)	(0.0035)	(0.0005)
Debt_gap	−0.0111***	−0.1296***	−0.0114***	0.0309***	−0.1744***	0.0371***
	(0.0009)	(0.0091)	(0.0009)	(0.0023)	(0.0108)	(0.0023)
Lev	0.0037***	0.1238***	0.0040***	0.0070**	0.2434***	−0.0017
	(0.0011)	(0.0146)	(0.0011)	(0.0028)	(0.0206)	(0.0027)
Roa	−0.0203***	0.0624***	−0.0201***	0.0321***	0.1221***	0.0278***
	(0.0026)	(0.0220)	(0.0026)	(0.0062)	(0.0360)	(0.0061)
CFO	−0.0041**	−0.1293***	−0.0044***	0.0171***	−0.1890***	0.0239***
	(0.0016)	(0.0163)	(0.0016)	(0.0040)	(0.0237)	(0.0040)
Dual	−0.0002	−0.0091**	−0.0003	−0.0008	−0.0089*	−0.0005
	(0.0003)	(0.0037)	(0.0003)	(0.0008)	(0.0047)	(0.0007)
ID	0.0010	0.0373	0.0011	−0.0068	−0.0194	−0.0061
	(0.0024)	(0.0278)	(0.0024)	(0.0061)	(0.0428)	(0.0057)
GDP	0.0123**	0.0803	0.0125**	0.0754***	0.1014	0.0718***
	(0.0050)	(0.0524)	(0.0050)	(0.0120)	(0.0681)	(0.0118)
_cons	0.0577***	−0.6024***	0.0561***	−0.0327**	−0.4562***	−0.0164
	(0.0053)	(0.0734)	(0.0053)	(0.0140)	(0.0853)	(0.0136)
Firm	Yes	Yes	Yes	Yes	Yes	Yes
Industry	Yes	Yes	Yes	Yes	Yes	Yes
N	14132	14132	14132	9836	9836	9836
Adj_R^2^	0.0881	0.0988	0.0887	0.0583	0.1252	0.0899

Note(s): *p < 0.1; **p < 0.05; ***p < 0.01; Robust standard errors in parentheses.

#### 4.3.6 Considering the impact of financial crisis and the COVID-19.

Due to the severe impact on the real economy during the global financial crisis in 2008 and the COVID-19 pandemic in 2020–2022, the impact of monetary policy on investment efficiency may be distorted. Therefore, a robustness test is conducted after excluding the samples from 2007–2009 and 2020–2022. The results in Table 12 show that the significance and direction of the coefficients of the monetary policy variable and the corporate debt maturity structure variable are consistent with the baseline regression, indicating that the role of monetary policy in corporate investment efficiency is very robust and its effect is not affected by extreme events.

**Table 12:** Robustness test: Kick out the samples during the financial crisis and the COVID-19.

	Under-investment			Over-investment		
	(1)	(2)	(3)	(4)	(5)	(6)
	UnderINV	Ldebt	UnderINV	OverINV	Ldebt	OverINV
MP	−0.0077***	0.1892***	−0.0073***	0.0213***	0.3009***	0.0114*
	(0.0024)	(0.0279)	(0.0024)	(0.0063)	(0.0402)	(0.0062)
Ldebt			−0.0021**			0.0329***
			(0.0011)			(0.0025)
Size	−0.0023***	0.0284***	−0.0023***	0.0011**	0.0202***	0.0004
	(0.0002)	(0.0025)	(0.0002)	(0.0005)	(0.0031)	(0.0005)
Debt_gap	−0.0103***	−0.1290***	−0.0106***	0.0303***	−0.1702***	0.0359***
	(0.0008)	(0.0081)	(0.0008)	(0.0021)	(0.0099)	(0.0021)
Lev	0.5706	24.4605	0.6226	1.4780	0.2337***	−0.0588
	(0.1952)	(2.6357)	(0.1969)	(0.5168)	(0.0185)	(0.5056)
Roa	−0.0201***	0.0608***	−0.0200***	0.0315***	0.1074***	0.0280***
	(0.0023)	(0.0194)	(0.0023)	(0.0056)	(0.0324)	(0.0055)
CFO	−0.0034**	−0.1329***	−0.0036***	0.0163***	−0.1906***	0.0226***
	(0.0014)	(0.0146)	(0.0014)	(0.0037)	(0.0220)	(0.0036)
Dual	−0.0003	−0.0069**	−0.0003	−0.0007	−0.0068	−0.0004
	(0.0003)	(0.0033)	(0.0003)	(0.0007)	(0.0042)	(0.0007)
ID	0.0024	0.0063	0.0024	−0.0063	−0.0485	−0.0047
	(0.0021)	(0.0249)	(0.0021)	(0.0055)	(0.0384)	(0.0053)
GDP	0.0190***	0.1045**	0.0193***	0.0706***	0.0936	0.0675***
	(0.0045)	(0.0472)	(0.0045)	(0.0105)	(0.0612)	(0.0103)
_cons	0.0586***	−0.6133***	0.0573***	−0.0240*	−0.4865***	−0.0080
	(0.0048)	(0.0645)	(0.0049)	(0.0123)	(0.0756)	(0.0121)
Firm	Yes	Yes	Yes	Yes	Yes	Yes
Industry	Yes	Yes	Yes	Yes	Yes	Yes
N	17183	17183	17183	11971	11971	11971
Adj_R^2^	0.0892	0.0986	0.0896	0.0580	0.1234	0.0861

Note(s): *p  <  0.1; **p  <  0.05; ***p  <  0.01; Robust standard errors in parentheses.

### 4.4 Analysis of heterogeneity

Based on the theoretical analysis in Section 2, this subsection launches the heterogeneity test with regional bank competition and financing constraints as heterogeneous variables. Attention is paid to whether the empirical results are consistent with hypotheses 4 and 5 to further verify the existence and rationality of the debt maturity effect of monetary policy.

#### 4.4.1 Bank competition.

The measure of regional bank competition level (CR5) refers to the practice of Jiang *et al.* [50], using the financial license information of the China Banking Regulatory Commission about bank institutions, to calculate the number of branches of each bank in each province in each year, and the proportion of the top five banks’ branches (CR5) measures the competition intensity of the banking industry in each city. The value of CR5 is between 0 and 1, and the smaller the value, the greater the degree of regional bank competition.


$$CR5=({Branch}_{1th}+{Branch}_{2th}+{Branch}_{3th}+{Branch}_{4th}+{Branch}_{5th})/{Total}_{Branches}$$ (10)

Where ${Branch}_{1th}$, ${Branch}_{2th}$, ${Branch}_{3th}$, ${Branch}_{4th}$ and ${Branch}_{5th}$ are the sum of the number of institutions of the five banks with the most branches in the province, ${Total}_{Branches}$ is the total number of branches of all banks in the province.

Divided into high bank competition areas and low bank competition areas by the average value. The empirical results in Tables 13 and 14 show that under the environment of loose monetary policy, in areas of high bank competition, loose monetary policy has a significant effect on corporate investment efficiency through the debt maturity structure channel, effectively mitigating corporate under-investment and promoting corporate over-investment. In regions with low bank competition, the debt maturity structure effect of loose monetary policy has a significant contribution to corporate over-investment behavior and no significant impact on corporate under-investment. This shows that under the environment of loose monetary policy, regardless of the intensity of bank competition, banks still prefer to provide funds to enterprises with excessive investment behavior rather than to enterprises with insufficient investment. Only in areas with a high degree of bank competition can banks be stimulated to increase financial support for enterprises with insufficient investment. The effect of the bank competition level on governing enterprise over-investment behavior is weaker than governing enterprise under-investment.The empirical results support hypothesis 4.

**Table 13:** Bank competition heterogeneity: high bank competition.

	Under-investment			Over-investment		
	(1)	(2)	(3)	(4)	(5)	(6)
	UnderINV	Ldebt	UnderINV	OverINV	Ldebt	OverINV
MP	−0.0099***	0.1759***	−0.0092***	0.0282***	0.2397***	0.0205**
	(0.0030)	(0.0330)	(0.0030)	(0.0081)	(0.0502)	(0.0080)
Ldebt			−0.0038**			0.0320***
			(0.0016)			(0.0035)
Size	−0.0023***	0.0335***	−0.0022***	0.0015**	0.0247***	0.0007
	(0.0003)	(0.0034)	(0.0003)	(0.0007)	(0.0042)	(0.0006)
Debt_total_gap	−0.0100***	−0.1188***	−0.0105***	0.0326***	−0.1519***	0.0374***
	(0.0011)	(0.0102)	(0.0011)	(0.0027)	(0.0125)	(0.0027)
Lev	0.0024*	0.1160***	0.0029**	0.0064*	0.2323***	−0.0010
	(0.0014)	(0.0185)	(0.0014)	(0.0035)	(0.0232)	(0.0034)
Roa	−0.0180***	0.0301	−0.0179***	0.0265***	0.0600	0.0246***
	(0.0031)	(0.0262)	(0.0031)	(0.0078)	(0.0417)	(0.0076)
CFO	−0.0043**	−0.1367***	−0.0049***	0.0167***	−0.1738***	0.0223***
	(0.0019)	(0.0181)	(0.0019)	(0.0049)	(0.0265)	(0.0048)
Dual	−0.0002	−0.0036	−0.0002	−0.0003	−0.0028	−0.0002
	(0.0004)	(0.0043)	(0.0004)	(0.0010)	(0.0056)	(0.0010)
ID	0.0008	−0.0003	0.0008	0.0007	−0.0856*	0.0034
	(0.0030)	(0.0312)	(0.0030)	(0.0081)	(0.0470)	(0.0079)
GDP	0.0270***	0.2194***	0.0278***	0.0816***	0.2269**	0.0743***
	(0.0068)	(0.0687)	(0.0068)	(0.0160)	(0.1005)	(0.0157)
_cons	0.0611***	−0.7386***	0.0583***	−0.0325**	−0.5419***	−0.0152
	(0.0060)	(0.0796)	(0.0060)	(0.0158)	(0.1002)	(0.0153)
Firm	Yes	Yes	Yes	Yes	Yes	Yes
Industry	Yes	Yes	Yes	Yes	Yes	Yes
N	9913	9913	9913	7203	7203	7203
Adj_R^2^	0.0632	0.0928	0.0644	0.0640	0.1159	0.0888

Note(s): *p < 0.1; **p < 0.05; ***p < 0.01;Robust standard errors in parentheses.

**Table 14:** Bank competition heterogeneity: Low bank competition.

	Under-investment			Over-investment		
	(1)	(2)	(3)	(4)	(5)	(6)
	UnderINV	Ldebt	UnderINV	OverINV	Ldebt	OverINV
MP	−0.0063	0.2046***	−0.0062	0.0307**	0.3673***	0.0179
	(0.0048)	(0.0503)	(0.0048)	(0.0131)	(0.0767)	(0.0127)
Ldebt			−0.0006			0.0346***
			(0.0020)			(0.0044)
Size	−0.0027***	0.0264***	−0.0027***	0.0033***	0.0160**	0.0028***
	(0.0003)	(0.0048)	(0.0003)	(0.0009)	(0.0073)	(0.0009)
Debt_total_gap	−0.0096***	−0.1391***	−0.0096***	0.0261***	−0.1916***	0.0328***
	(0.0014)	(0.0153)	(0.0015)	(0.0039)	(0.0201)	(0.0041)
Lev	0.0002	0.1395***	0.0003	0.3922	48.5305	−1.2881
	(0.0019)	(0.0228)	(0.0019)	(0.9976)	(7.7035)	(0.9789)
Roa	−0.0313***	0.1218***	−0.0312***	0.0357***	0.2239***	0.0279**
	(0.0044)	(0.0379)	(0.0044)	(0.0123)	(0.0673)	(0.0121)
CFO	−0.0018	−0.1232***	−0.0019	0.0189***	−0.2034***	0.0260***
	(0.0024)	(0.0280)	(0.0025)	(0.0071)	(0.0453)	(0.0070)
Dual	−0.0001	−0.0019	−0.0001	−0.0015	−0.0230***	−0.0007
	(0.0006)	(0.0067)	(0.0006)	(0.0016)	(0.0082)	(0.0016)
ID	0.0015	0.0538	0.0015	−0.0224**	−0.0487	−0.0207**
	(0.0040)	(0.0471)	(0.0040)	(0.0095)	(0.0871)	(0.0090)
GDP	0.0116	0.1514	0.0117	0.0757***	0.0167	0.0751***
	(0.0089)	(0.1006)	(0.0089)	(0.0242)	(0.1277)	(0.0240)
_cons	0.0723***	−0.6054***	0.0719***	−0.0621***	−0.3484**	−0.0500**
	(0.0080)	(0.1125)	(0.0080)	(0.0213)	(0.1687)	(0.0204)
Firm	Yes	Yes	Yes	Yes	Yes	Yes
Industry	Yes	Yes	Yes	Yes	Yes	Yes
N	5713	5713	5713	3624	3624	3624
Adj_R^2^	0.0858	0.0830	0.0857	0.0479	0.1067	0.0800

Note(s): *p < 0.1; **p < 0.05; ***p < 0.01;Robust standard errors in parentheses.

#### 4.4.2 financing constraints.

For the measure of financing constraints, we draw on the methodology of Hadlock and Pierce [51]. The calculation process is as follows: (1) Standardize the three variables of firm size, age, and cash dividend payout ratio by year, and determine the dummy variable of financing constraints QUFC according to the standardized mean values of the variables: firms with mean values higher than one-third of the quartile are less constrained, and QUFC is 0; firms with mean values lower than one-third of the quartile are more constrained, and QUFC is 1; (2) Fit a Logit model to the firm’s financing constraints in each year. The corresponding QUFC takes 1; (3) use Logit model to fit the probability of occurrence of financing constraints of enterprises in each year, and define it as the financing constraints index FC (with the value between 0 and 1), the larger FC is, the more serious the financing constraints problem of enterprises is. CASHDIV in the model (12) denotes cash dividends declared in the year, ta denotes total assets, NWC denotes net working capital, and EBIT denotes earnings before interest and taxes.


$$P(QUFC=1|Z_{it})=e^{Z_{it}}/(1+e^{Z_{it}})\mathrm{\backslash ]}$$ (11)

$$Z_{it}=\alpha_{0}+\alpha_{1}{Size}_{it}+\alpha_{2}{Lev}_{it}+\alpha_{3}{\left(CASHDIV/ta\right)}_{it}+\alpha_{4}{MB}_{it}+\alpha_{5}{\left(NWC/ta\right)}_{it}+\alpha_{6}{\left(EBIT/ta\right)}_{it}\mathrm{\backslash ]}$$ (12)

High and low financing constrained firms are categorized by the mean value of financing constraints. Tables 15 and 16 report the results of the heterogeneity test for financing constraints. The results in Table 15 show that for high financing constraint firms, accommodative monetary policy mitigates firms’ under-investment, but the monetary policy debt maturity structure effect does not play a role. Loose monetary policy also has no significant effect on over-investment by firms with high financing constraints. The results in Table 15 show that for firms with low financing constraints, the monetary policy debt maturity structure channel works effectively to significantly mitigate firms’ under-investment and promote firms’ over-investment. Therefore, the empirical results are consistent with the expectation of hypothesis 5 and further support the existence and rationality of the debt maturity structure channel of monetary policy.

**Table 15:** Heterogeneity of financing constraints: high financing constraints.

	Under-investment			Over-investment		
	(1)	(2)	(3)	(4)	(5)	(6)
	UnderINV	Ldebt	UnderINV	OverINV	Ldebt	OverINV
MP	−0.0080**	0.0804**	−0.0080**	−0.0051	0.2006***	−0.0111
	(0.0036)	(0.0344)	(0.0036)	(0.0098)	(0.0604)	(0.0094)
Ldebt			−0.0001			0.0299***
			(0.0017)			(0.0041)
Size	−0.0027***	0.0380***	−0.0027***	0.0000	0.0347***	−0.0010
	(0.0003)	(0.0039)	(0.0004)	(0.0008)	(0.0052)	(0.0008)
Debt_total_gap	−0.0101***	−0.1347***	−0.0101***	0.0188***	−0.1675***	0.0238***
	(0.0013)	(0.0118)	(0.0013)	(0.0031)	(0.0154)	(0.0032)
Lev	0.0035**	0.1488***	0.0035**	0.0054	0.2441***	−0.0019
	(0.0015)	(0.0160)	(0.0015)	(0.0037)	(0.0244)	(0.0038)
Roa	−0.0135***	−0.0009	−0.0135***	0.0258***	0.0428	0.0245***
	(0.0034)	(0.0251)	(0.0034)	(0.0081)	(0.0429)	(0.0081)
CFO	−0.0054**	−0.1072***	−0.0055**	0.0110**	−0.1597***	0.0158***
	(0.0022)	(0.0211)	(0.0022)	(0.0055)	(0.0292)	(0.0055)
Dual	0.0002	−0.0071*	0.0002	−0.0007	−0.0078	−0.0005
	(0.0004)	(0.0043)	(0.0004)	(0.0010)	(0.0059)	(0.0010)
ID	0.0008	−0.0065	0.0008	−0.0099	0.0341	−0.0109
	(0.0035)	(0.0332)	(0.0035)	(0.0082)	(0.0513)	(0.0080)
GDP	0.0179**	−0.0004	0.0179**	0.0222	−0.2138**	0.0286*
	(0.0074)	(0.0656)	(0.0074)	(0.0160)	(0.0877)	(0.0158)
_cons	0.0682***	−0.7986***	0.0682***	0.0151	−0.7500***	0.0375**
	(0.0079)	(0.0857)	(0.0080)	(0.0187)	(0.1179)	(0.0182)
Firm	Yes	Yes	Yes	Yes	Yes	Yes
Industry	Yes	Yes	Yes	Yes	Yes	Yes
N	8934	8934	8934	5903	5903	5903
Adj_R^2^	0.0446	0.1028	0.0445	0.0227	0.1481	0.0468

Note(s): *p < 0.1; **p < 0.05; ***p < 0.01;Robust standard errors in parentheses.

**Table 16:** Heterogeneity of financing constraints: high financing constraints.

	Under-investment			Over-investment		
	(1)	(2)	(3)	(4)	(5)	(6)
	UnderINV	Ldebt	UnderINV	OverINV	Ldebt	OverINV
MP	−0.0071**	0.3460***	−0.0062*	0.0343***	0.3872***	0.0211**
	(0.0035)	(0.0434)	(0.0035)	(0.0092)	(0.0533)	(0.0092)
Ldebt			−0.0025*			0.0340***
			(0.0015)			(0.0035)
Size	−0.0024***	0.0290***	−0.0023***	0.0011*	0.0211***	0.0004
	(0.0003)	(0.0041)	(0.0003)	(0.0007)	(0.0050)	(0.0006)
Debt_total_gap	−0.0096***	−0.1341***	−0.0099***	0.0401***	−0.1666***	0.0457***
	(0.0011)	(0.0113)	(0.0011)	(0.0031)	(0.0139)	(0.0031)
Lev	0.0003	0.1008***	0.0006	0.0069	0.2279***	−0.0008
	(0.0017)	(0.0251)	(0.0017)	(0.0045)	(0.0329)	(0.0043)
Roa	−0.0301***	0.1073***	−0.0298***	0.0415***	0.1835***	0.0353***
	(0.0035)	(0.0342)	(0.0035)	(0.0091)	(0.0504)	(0.0089)
CFO	0.0006	−0.1799***	0.0001	0.0214***	−0.2444***	0.0297***
	(0.0018)	(0.0210)	(0.0018)	(0.0058)	(0.0312)	(0.0057)
Dual	−0.0007*	−0.0116**	−0.0007*	−0.0008	−0.0126*	−0.0003
	(0.0004)	(0.0050)	(0.0004)	(0.0011)	(0.0065)	(0.0010)
ID	0.0043	−0.0120	0.0042	−0.0087	−0.0815	−0.0059
	(0.0029)	(0.0343)	(0.0029)	(0.0082)	(0.0536)	(0.0078)
GDP	0.0162***	0.1804***	0.0167***	0.0918***	0.3082***	0.0813***
	(0.0061)	(0.0663)	(0.0061)	(0.0157)	(0.0856)	(0.0155)
_cons	0.0638***	−0.6487***	0.0622***	−0.0248	−0.4826***	−0.0084
	(0.0069)	(0.0981)	(0.0069)	(0.0166)	(0.1190)	(0.0160)
Firm	Yes	Yes	Yes	Yes	Yes	Yes
Industry	Yes	Yes	Yes	Yes	Yes	Yes
N	8249	8249	8249	6068	6068	6068
Adj_R^2^	0.0847	0.0789	0.0852	0.0958	0.1024	0.1225

Note(s): *p < 0.1; **p < 0.05; ***p < 0.01;Robust standard errors in parentheses.

## 5. Conclusion

Based on the data of China’s A-share listed companies from 2007 to 2022, this paper conducts a research based on the path of “monetary policy-debt maturity structure-corporate investment efficiency”. It is found that loose monetary policy can have a dual effect on corporate investment efficiency by extending corporate debt maturity structure, which is manifested in alleviating corporate under-investment and promoting corporate over-investment. The above conclusions are still valid after the robustness tests such as modifying the model setting, placebo test, substitution of variable measures, and considering the effects of special periods and special regions. Heterogeneity analysis shows that in high bank competition areas, the debt maturity structure effect of monetary policy is effective in mitigating under-investment and promoting over-investment, while in low bank competition areas, the debt maturity structure effect of monetary policy promotes over-investment but does not have a significant effect on under-investment. In addition, the debt maturity structure effect of monetary policy can effectively alleviate under-investment and promote over-investment of enterprises with low financing constraints, while it has no significant effect on the under-investment and over-investment behavior of enterprises with high financing constraints. The research in this paper reveals a new mechanism by which monetary policy affects the efficiency of corporate investment.

The research of this paper has certain theoretical value and practical significance. The most prominent theoretical value of this paper is that it enriches the research on the credit transmission mechanism of monetary policy. Most of the existing studies focus on the liquidity effect of monetary policy on the real economy from the perspective of credit aggregates. This paper starts from the perspective of debt maturity structure, focusing on the existence of the debt maturity structure effect of monetary policy and its effect. The theoretical findings of the debt maturity structure channel of monetary policy provide guidance for the central bank to formulate monetary policy, which is of strong practical significance. Based on the findings of heterogeneity in this paper, we can find that the effect of aggregate monetary policy is affected by the characteristics of banks and enterprises themselves, and the final effect may not be ideal. As far as this paper is concerned, the main purpose of loose monetary policy should be to provide funds to firms that lack funds and reduce under-investment behavior. However, we find that banks do not prioritize the flow of funds to these types of firms, and more funds flow to overinvested firms that are relatively well-funded, further exacerbating over-investment by firms, reducing investment efficiency, and resulting in a waste of resources. Therefore, an important practical inspiration of this paper is to implement a targeted structural monetary policy in order to better serve the real economy.

This paper expands the study of the economic consequences of monetary policy, emphasizes the important role of debt maturity structure in the transmission process of monetary policy, and puts forward the following policy recommendations based on the findings of this paper.

First, pay attention to the regulation of monetary policy on debt quantity and debt structure at the same time. Focus on the innovation and implementation of structural monetary policy, such as targeted long-term monetary policy support tools, in order to realize the precise support of monetary policy to the specific aspects of the real economy and improve the effectiveness of monetary policy.

Second, we should focus on the guidance of bank credit investment, and guide banks to surrender funds to underinvested enterprises that are more in need of financial support through bank performance appraisals and other means.

Third, encourage bank competition to stimulate the vitality of the industry. At the regulatory level, liberalize market access, lower the threshold for new banks to enter the market, and allow more banks to enter the market. At the same time, through the formulation and improvement of relevant laws and regulations, the market competition behavior of banks is regulated. In terms of operation, banks are encouraged to differentiate their operations according to their own resources and advantages to form their own characteristics and competitive advantages. For example, some banks can focus on serving small and micro enterprises, agriculture and other areas. The scope of the banking system’s service recipients is constantly being expanded to better serve the real economy.

## Supporting information

S1 Supporting information. (RAR)

## References

[1] Stein JC. Agency, Information and Corporate Investment. Handbook of the Economics of Finance. Elsevier. 2003. p. 111–65. doi: 10.1016/s1574-0102(03)01006-9

[2] Yang Z, Lu Y, Tan W. Monetary policy tightening, accounting information comparability, and underinvestment: Evidence from China. Economic Analysis and Policy. 2021;70:123–47. doi: 10.1016/j.eap.2021.02.005

[3] Zhang M, Liu Z. Balance sheet recession: theoretical framework, Japan’s experience, and China’s challenges. Comparative Economic and Social Systems. 2024;:99–106.

[4] Tian D. Revisionist neoliberalism: The new strategy of the British government. Chinese Journal of European Studies. 2020;38:110–34.

[5] Ouyang X. The Belt and Road initiative and China-Russia relations. International Economic Review. 2017;5(6):49–61.

[6] Li S, Wang Y. Budget deficit, federal debt, and the concomitant fights between the Democrats and Republicans in the U.S. The Chinese Journal of American Studies. 2024;38:55–77.

[7] Myers SC. Determinants of corporate borrowing. Journal of Financial Economics. 1977;5(2):147–75. doi: 10.1016/0304-405x(77)90015-0

[8] Naeem K, Li MC. Corporate investment efficiency: The role of financial development in firms with financing constraints and agency issues in OECD non-financial firms. International Review of Financial Analysis. 2019;62:53–68. doi: 10.1016/j.irfa.2019.01.003

[9] Chen Z, Jiang K. Digitalization and corporate investment efficiency: Evidence from China. Journal of International Financial Markets, Institutions and Money. 2024;91:101915. doi: 10.1016/j.intfin.2023.101915

[10] Ren Y, Liu X, Zhu Y. Can the development of digital finance and information transparency improve enterprise investment efficiency?. Finance Research Letters. 2025;73:106597. doi: 10.1016/j.frl.2024.106597

[11] He Y, Chen C, Hu Y. Managerial overconfidence, internal financing, and investment efficiency: Evidence from China. Research in International Business and Finance. 2019;47:501–10. doi: 10.1016/j.ribaf.2018.09.010

[12] Tran Phuong T, Le A-T, Ouyang P. Board tenure diversity and investment efficiency: A global analysis. Journal of International Financial Markets, Institutions and Money. 2022;81:101657. doi: 10.1016/j.intfin.2022.101657

[13] Bilyay-Erdogan S, Danisman GO, Demir E. ESG performance and investment efficiency: The impact of information asymmetry. Journal of International Financial Markets, Institutions and Money. 2024;91:101919. doi: 10.1016/j.intfin.2023.101919

[14] Khan MK, He Y, Akram U, Sarwar S. Financing and monitoring in an emerging economy: Can investment efficiency be increased?. China Economic Review. 2017;45:62–77. doi: 10.1016/j.chieco.2017.05.012

[15] Wan J, Lee C-C. Corporate investment and the dilemma of the monetary policy: Evidence from China. Economic Analysis and Policy. 2023;78:106–21. doi: 10.1016/j.eap.2023.03.002

[16] Wang Y, Cui R, Gao H, Lu X, Hu X. Can the construction of the social credit system improve the efficiency of corporate investment?. International Review of Economics & Finance. 2024;96:103510. doi: 10.1016/j.iref.2024.103510

[17] Chen F, Hope OK, Li Q, Wang X. Financial reporting quality and investment efficiency of private firms in emerging markets. The Accounting Review. 2011;86:1255–88.

[18] Riccetti L, Russo A, Gallegati M. Financial Regulation in an Agent-Based Macroeconomomic Model. SSRN Journal. 2013. doi: 10.2139/ssrn.2345904

[19] Li X-L, Xie P, Ding H, Si D-K. Central bank lending facility and investment efficiency of non-SOEs: evidence from China. Economic Modelling. 2023;126:106421. doi: 10.1016/j.econmod.2023.106421

[20] Zhong K, Cheng X, Zhang W. The moderate adjustment of monetary policy and the phenomenon of corporate long-term investment with short-term financing. Management World. 2016;:87-98 114 188. doi: 10.19744/j.cnki.11-1235/f.2016.03.008

[21] Cutillas Gomariz MF, Sánchez Ballesta JP. Financial reporting quality, debt maturity and investment efficiency. Journal of Banking & Finance. 2014;40:494–506. doi: 10.1016/j.jbankfin.2013.07.013

[22] Sun H, Zheng L, Liao J. Monetary policy, credit term structure and the corporate financialization. Studies of International Finance. 2021;:13–21. doi: 10.16475/j.cnki.1006-1029.2021.08.002

[23] Jensen MC, Meckling WH. Theory of the firm: managerial behavior, agency costs and ownership structure. Journal of Financial Economics. 1976;3:305–60.

[24] Jensen MC. Agency costs of free cash flow, corporate finance, and takeovers. Corporate Bankruptcy. Cambridge University Press. 1996. p. 11–6. doi: 10.1017/cbo9780511609435.005

[25] Kane A, Marcus AJ, McDonald RL. Debt policy and the rate of return premium to leverage. Journal of Financial and Quantitative Analysis. 1985;20:479–99.

[26] Kale JR, Noe TH. Risky Debt Maturity Choice In A Sequential Game Equilibrium. J of Financial Research. 1990;13(2):155–66. doi: 10.1111/j.1475-6803.1990.tb00545.x

[27] Diamond DW. Debt Maturity Structure and Liquidity Risk. The Quarterly Journal of Economics. 1991;106(3):709–37. doi: 10.2307/2937924

[28] Kirch G, Terra PRS. Determinants of corporate debt maturity in South America: Do institutional quality and financial development matter?. Journal of Corporate Finance. 2012;18(4):980–93. doi: 10.1016/j.jcorpfin.2012.05.004

[29] Keefe MO, Nguyen PH. The influence of cash flow volatility on firm use of debt of different maturities or zero-debt: International evidence. International Review of Economics & Finance. 2023;86:684–700. doi: 10.1016/j.iref.2023.03.035

[30] Nguyen VH, Choi B, Agbola FW. Corporate social responsibility and debt maturity: Australian evidence. Pacific-Basin Finance Journal. 2020;62:101374. doi: 10.1016/j.pacfin.2020.101374

[31] Tran DTT, Phan HV. Government economic policy uncertainty and corporate debt contracting. Int Rev Finance. 2021;22(1):169–99. doi: 10.1111/irfi.12347

[32] Borio C, Zhu H. Capital regulation, risk-taking and monetary policy: A missing link in the transmission mechanism?. Journal of Financial Stability. 2012;8:236–51.

[33] Delis MD, Kouretas GP. Interest rates and bank risk-taking. Journal of Banking & Finance. 2011;35(4):840–55. doi: 10.1016/j.jbankfin.2010.09.032

[34] Li B, Chen Y, Li Y. An empirical study on the relationship between managerial entrenchment, R&D investment, and corporate governance. Science Research Management. 2014;35:99–106. doi: 10.19571/j.cnki.1000-2995.2014.07.013

[35] Wang K, Liu J, Li X. Industrial policy, government support and corporate investment efficiency. Management World. 2017;:113-124 145 188. doi: 10.19744/j.cnki.11-1235/f.2017.03.008

[36] Narayanan MP. Managerial Incentives for Short‐term Results. The Journal of Finance. 1985;40(5):1469–84. doi: 10.1111/j.1540-6261.1985.tb02395.x

[37] Ben-David I, Graham J, Harvey C. Managerial overconfidence and corporate policies. Cambridge, MA: National Bureau of Economic Research Report No.: w13711.; 2007 Dec. p. w13711. doi: 10.3386/w13711

[38] Barclay MJ, Smith JR CW. The Maturity Structure of Corporate Debt. The Journal of Finance. 1995;50(2):609–31. doi: 10.1111/j.1540-6261.1995.tb04797.x

[39] Han G, Zhao G. The interactive influence of accounting information quality and debt maturity structure on investment efficiency. Wuhan University Journal(Philosophy & Social Sciences). 2016;69: 58–65. 10.14086/j.cnki.wujss.2016.04.007

[40] Judd CM, Kenny DA. Process Analysis. Eval Rev. 1981;5(5):602–19. doi: 10.1177/0193841x8100500502

[41] Baron RM, Kenny DA. The moderator-mediator variable distinction in social psychological research: conceptual, strategic, and statistical considerations. J Pers Soc Psychol. 1986;51(6):1173–82. doi: 10.1037//0022-3514.51.6.1173 3806354

[42] Wen Z, Chang L, Hau KT, Liu H. Testing and application of the mediating effects. Acta Psychologica Sinica. 2004;36:614.

[43] Zheng L, Jia Liao YY, Mo B, Liu Y. The effect credit term structure of monetary policy on firms’ “short-term debt for long-term investment” behavior: empirical evidence from China. Electron Res Arch. 2023;31:1498–523.

[44] Richardson S. Over-investment of free cash flow. Rev Acc Stud. 2006;11(2–3):159–89. doi: 10.1007/s11142-006-9012-1

[45] Huang F, Ge L, Wu S. Minority shareholder protection, corporate governance, and investment efficiency. Finance Research Letters. 2023;58:104506. doi: 10.1016/j.frl.2023.104506

[46] Hu J, Kong M, Wang K. The influencial factors and economic consequences of corporate investment efficiency: A literature review. 2022 7th International Conference on Financial Innovation and Economic Development (ICFIED 2022). Atlantis Press; 2022. p. 2389–2394. https://www.atlantis-press.com/proceedings/icfied-22/125971933

[47] Wu JY, Opare S, Bhuiyan MdBU, Habib A. Determinants and consequences of debt maturity structure: A systematic review of the international literature. International Review of Financial Analysis. 2022;84:102423. doi: 10.1016/j.irfa.2022.102423

[48] Mao J, Guan X. Study on the effects of local governments’ enterprise-helping relief policies: Evidence from listed companies. Economic Research Journal. 2022;57:82–98.

[49] Biddle GC, Hilary G, Verdi RS. How does financial reporting quality relate to investment efficiency?. Journal of Accounting and Economics. 2009;48(2–3):112–31. doi: 10.1016/j.jacceco.2009.09.001

[50] Jiang F, Cai W, Cai X, Li X. Microeconomic effects of bank competition: Evidence from corporate financial constraints. Economic Research Journal. 2019;54:72–88.

[51] Hadlock CJ, Pierce JR. New evidence on measuring financial constraints: moving beyond the KZ index. The Review of Financial Studies. 2010;23:1909–40.

